# Nanorobot-Cell Communication via *In Situ* Generation of Biochemical Signals: Toward Regenerative Therapies

**DOI:** 10.1021/acsnano.5c02092

**Published:** 2025-06-17

**Authors:** Roshan Velluvakandy, Xiaohui Ju, Martin Pumera

**Affiliations:** † Future Energy and Innovation Laboratory, Central European Institute of Technology, 48274 Brno University of Technology, Purkyňova 123, 61200 Brno, Czech Republic; ‡ Advanced Nanorobots & Multiscale Robotics Laboratory, Faculty of Electrical Engineering and Computer Science, VSB - Technical University of Ostrava, 17. listopadu 2172/15, 70800 Ostrava, Czech Republic; § Department of Medical Research, China Medical University Hospital, China Medical University, No. 91 Hsueh-Shih Road, 40402 Taichung, Taiwan

**Keywords:** nanorobot, enhanced diffusion, communication, cell signaling, steady-state

## Abstract

Achieving precise control of cellular processes drives possibilities for next-generation therapeutic approaches. However, existing technologies for influencing cell behavior primarily rely on specific drug delivery, limiting their ability to mimic natural cellular communication processes. In this work, we developed glucose-powered gold-silica (Au-SiO_2_) nanorobots that induce cell migration by generating steady-state hydrogen peroxide (H_2_O_2_) as a biochemical signaling molecule to mimic natural cellular communication with high spatial resolution. These nanorobots leverage the unique 2-in-1 catalytic activity of gold nanoparticles for glucose oxidation and H_2_O_2_ decomposition, allowing for precise control over the generation of steady-state H_2_O_2_ concentration and enhanced diffusion powered by glucose within the cellular microenvironment. We further demonstrated that at low dosages of nanorobots, the steady-state H_2_O_2_ generation promotes cell migration and proliferation, while higher dosages of nanorobots slow down cell proliferation. The proposed design of this biocompatible nanorobot is intended to enable communication with the environment and provide a noninvasive, biochemical command system for regulating cellular behavior. Additionally, we show proof of principle of a method by which nanorobots can augment wound healing and similar regenerative therapies.

## Graphical abstract

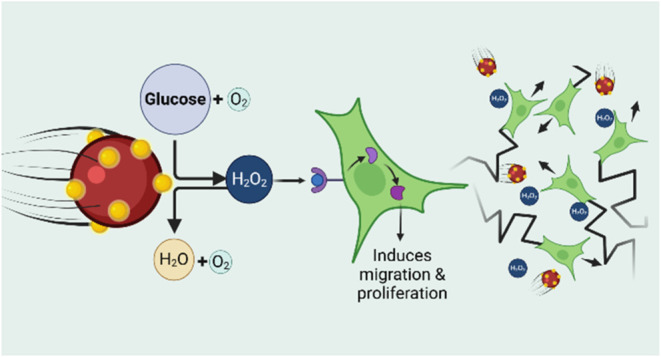

## Introduction

Over the past 20 years, the field of micro/nanorobotics has made remarkable progress, bringing us closer to a future where nanoscale machines could revolutionize fields such as medicine and environmental science. Early successes include micro/nanorobots capable of precise navigation within biological systems, transporting cargo, and targeted drug release at specific sites.^1–4^ However, to fully realize the potential of these technologies, nanorobots must evolve beyond these initial functions. The next frontier in the biomedical application of nanorobots is to empower them to actively regulate cellular behavior and engage with the cellular microenvironment. This requires a critical capability of nanorobots: the ability to “communicate” with the environment by sending and receiving signals that can influence cellular behavior in a controlled manner.^5^ This form of communication would allow nanorobots to regulate vital cellular activities and thus become active participants in biological processes. Communication between cells is typically carried out by chemical molecules that bind with specific receptors to activate a downstream signaling cascade. Given the complexity of cellular behavior, there exists a wide variety of signaling molecules, including but not limited to, ions, peptides, proteins, lipids, reactive oxygen species, nucleic acids, and even gases.^6^

Designing a nanorobot to communicate with cells requires the nanorobots to be able to either produce or respond appropriately to cellular signaling molecules. One of the biochemical signals that can influence cell behavior is H_2_O_2_, which functions both as a cellular metabolite and a signaling molecule that can influence processes such as cell growth, differentiation, and migration.^7–11^ Historically, as one of the reactive oxygen species (ROS), H_2_O_2_ has been viewed primarily as a toxic byproduct of cellular respiration that needed to be quickly eliminated. Its presence has been long associated with oxidative stress and cellular damage.^12^ However, research over the past two decades has dramatically shifted this perception.^7^ We now understand that H_2_O_2_ also plays an essential role in cellular communication and regulation.^13^

Nanorobots could exploit this signaling pathway by producing and releasing regulated quantity of H_2_O_2_ at precise locations to guide cell behavior, much like how natural signals work in the body. Producing H_2_O_2_ can be done trivially with the use of the enzyme glucose oxidase (GOx), an enzyme that catalyzes the oxidation of glucose to generate H_2_O_2_ as a byproduct.^14^ However, this approach presents a challenge in controlling the generated H_2_O_2_ concentration, as GOx continuously produces H_2_O_2_ as long as glucose is present. This would result in the generation of excessive H_2_O_2_, potentially causing oxidative damage to nearby tissues and disrupting the delicate balance needed for effective signaling. To address this issue, researchers have presented an enzyme combination approach using glucose oxidase and catalase (GOx/Cat).^15–18^ Catalase is an enzyme that can decompose hydrogen peroxide to water and oxygen. Combined use of GOx/Cat therefore allows for control over the rate of generation and decomposition of H_2_O_2_ maintaining a precise steady-state concentration of H_2_O_2_.^17,19,20^ This balanced production and degradation create an environment where H_2_O_2_ can serve as a controlled signal, rather than a toxic agent. While enzyme-based approaches, such as the GOx/Cat system, show promise, they also have drawbacks, including limited enzyme lifetimes and susceptibility to protease degradation. Here, nanozymes present a promising alternative in the form of a single-component system.^21^ In recent years, gold nanoparticles (AuNPs) have gained significant attention as nanozymes—nanomaterials that mimic the catalytic activities of natural enzymes. Gold nanoparticles, in particular, demonstrate catalytic activity comparable to enzymes glucose oxidase, catalase, peroxidase, and superoxide dismutase among others.^22,23^ The dual functionality of GOx and Cat mimicking activities of gold nanoparticles makes them highly promising for applications that require precise control over chemical reactions for the production and regulation of H_2_O_2_ in biological systems.

In this work, we present the design of glucose-powered gold-silica (Au-SiO_2_) nanorobots that exhibit enhanced diffusion and can further induce cell migration by generating a steady-state concentration of H_2_O_2_ as signaling molecules to mimic natural cellular communication (Figure 1). The nanorobot is capable of catalyzing both the oxidation of glucose to produce H_2_O_2_ and the subsequent decomposition of H_2_O_2_. The subtle balance between these two reactions results in enhanced glucose-powered diffusion and a controlled, steady-state concentration of H_2_O_2_, with the concentration directly proportional to the nanorobot concentration. *In vitro* experiments show that these nanorobots can further regulate cellular behavior such as migration and proliferation, utilizing the generated steady-state H_2_O_2_ as a biochemical signaling molecule in the surrounding cellular microenvironment. Apart from their implications in basic research, where these nanorobots enable the study of controlled H_2_O_2_ signals on cell behavior, this system also holds significant potential for applications such as precise wound healing and regenerative medicine.

**Figure 1:**
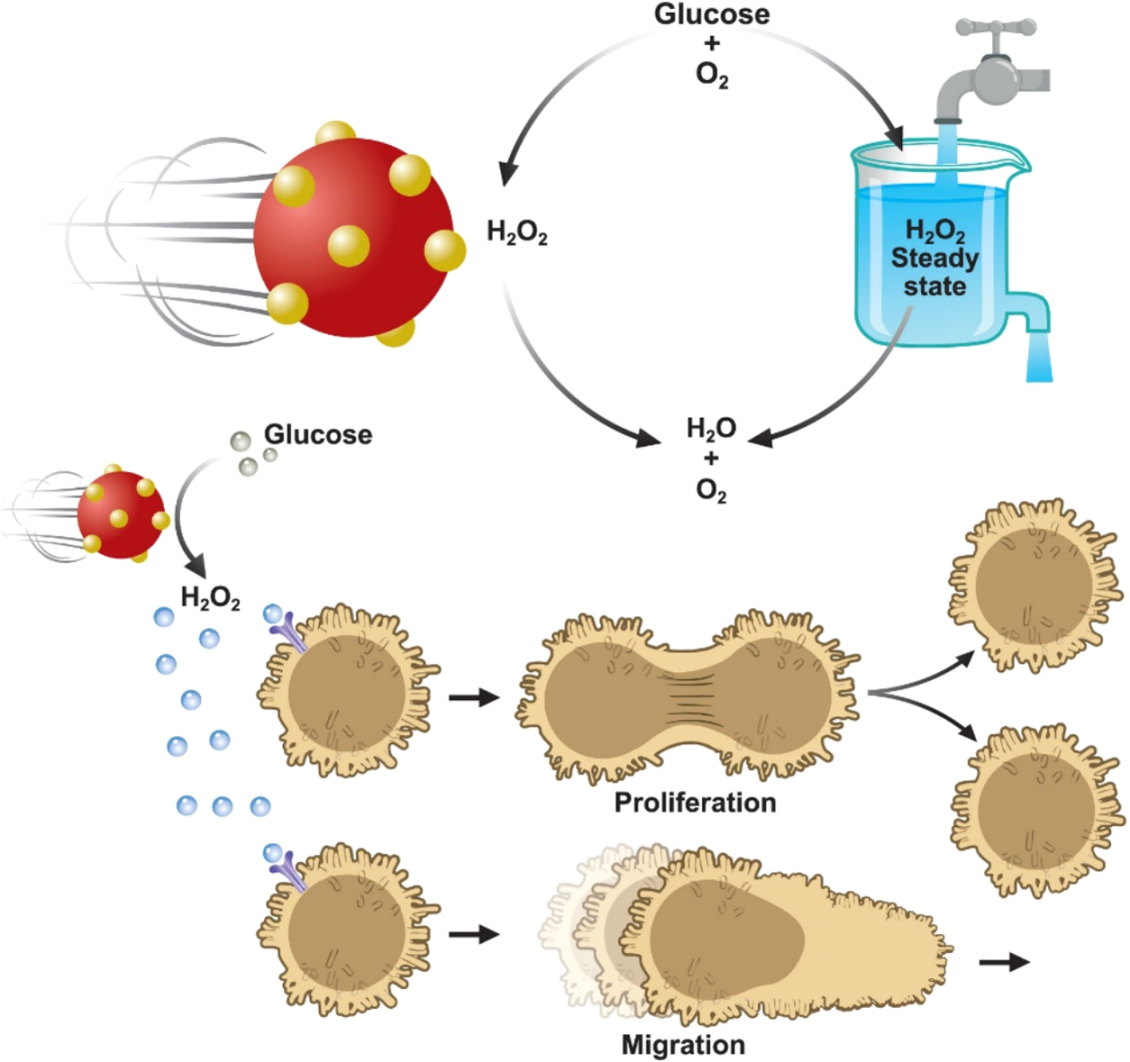
Schematic representation of using Au-SiO_2_ nanorobots to communicate with cells. Au-SiO_2_ nanorobots catalytic conversion of glucose to H_2_O_2_, followed by its decomposition to H_2_O and O_2_ leading to the steady-state generation of H_2_O_2_. Application of nanorobots for inducing cell migration, where the self-regulated production of H_2_O_2_ acts as a signaling molecule to induce cell proliferation and cell migration.

## Results and Discussion

### Synthesis and Characterization of Glucose-Powered Au-SiO_2_ Nanorobots

Glucose-powered Au-SiO_2_ nanorobots were constructed by the assembly of two main components: “naked” gold nanoparticles (Au NPs) and aminated (−NH_2_) silica nanoparticles (Figure 2A). The oxidation of glucose to gluconic acid has been first demonstrated with “naked” Au NPs of sizes under 10 nm.^24^ In this work, Au NPs were synthesized based on the direct reduction of gold­(III) chloride hydrate (HAuCl_4_) by sodium borohydride (NaBH_4_).^25^ The average size of synthesized Au NPs was tuned by optimizing the ratio of HAuCl_4_ and NaBH_4_. High-angle annular dark-field scanning transmission electron microscopy (HAADF-STEM) image shows that the synthesized Au NPs appear relatively well dispersed with an average diameter of 4.2 ± 0.7 nm (Figure 2B and E). The synthesis process did not involve any stabilizing agents like citrates or polymers; instead, the Au NPs were stabilized solely by ionic interaction with reactants such as Na^+^, BOH, BH_4_^–^, and Cl^–^ ions. Although the lack of capping agents can reduce the long-term stability of Au NPs, our objective is to produce “naked” gold NPs, which are expected to exhibit higher catalytic activity of glucose oxidation due to their exposed surface. Capping with different molecules has been shown to influence this GOx mimetic activity and in some cases completely block it.^26^

**Figure 2:**
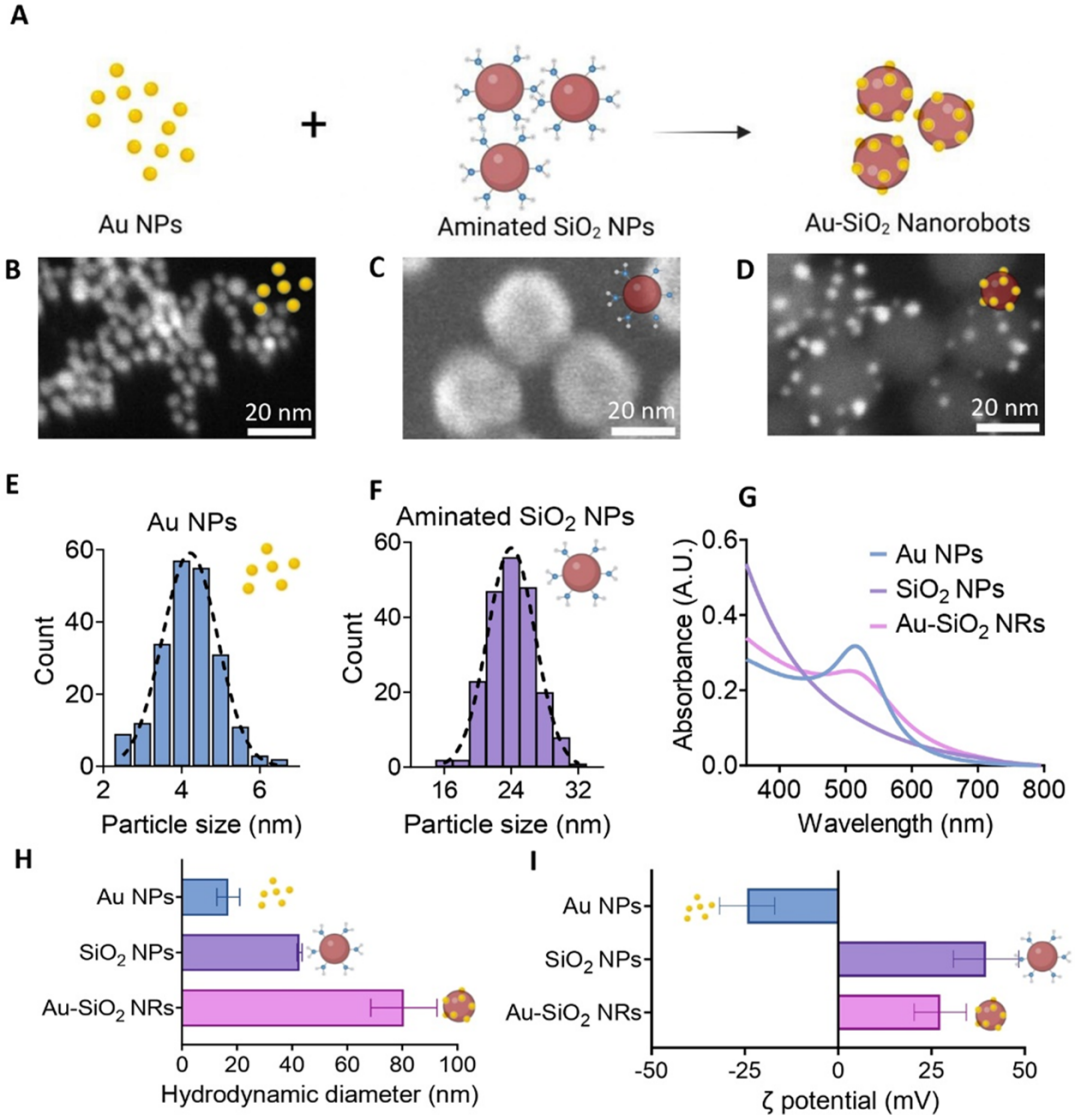
Material characterization demonstrating the successful synthesis of Au-SiO_2_ nanorobots. (A) Schematic representation of the construction steps of Au-SiO_2_ nanorobots (Au-SiO_2_ NRs). (B) STEM-HAADF image of Au NPs. (C) SEM image of aminated SiO_2_ particles. (D) STEM-HAADF image of Au-SiO_2_ nanorobots. (E) Particle size distribution of Au NPs (*N* > 200). (F) Particle size distribution of aminated SiO_2_ particles (*N* > 200). (G) Normalized absorbance in visible spectra by Au NPs, aminated SiO_2_ NPs, and Au-SiO_2_ nanorobots in colloidal solution. (H) Hydrodynamic size measured by DLS showing the change in size after Au NPs attachment; error bars represent standard deviation. (I) ζ potentials of Au NPs, SiO_2_ NPs, and Au-SiO_2_ nanorobots; error bars represent standard deviation. Schematic was created with BioRender.com.

The Au-SiO_2_ nanorobots (Au-SiO_2_ NRs) were assembled by attaching the dispersed Au NPs colloids onto the surface of the aminated SiO_2_ NPs. Commercial NH_2_–SiO_2_ NPs with a core size around 24.1 ± 2.7 nm characterized by scanning electron microscopy (SEM) (Figure 2C and F) were chosen since their surface is positively charged due to amination. We monitored the hydrodynamic diameter and surface ζ potential of the nanoparticles at each fabrication step using dynamic light scattering (DLS). The results reveal a hydrodynamic size increase after the assembly attributed to the addition of Au NPs on silica surfaces (Figure 2H). The changes of ζ potentials of measured samples followed the expected behavior during synthesis, with the final product reaching a value of 27.3 ± 6.9 mV (Figure 2I). Due to the difference in surface charges of the Au NPs and NH_2_–SiO_2_ NPs, the Au NPs electrostatically adsorbed onto the surface of the silica nanoparticles. This assembly was confirmed by the HAADF-STEM image shown in Figure 2D, where Au NPs randomly decorated the surface of the silica nanoparticles. The presence of Au NPs on the Au-SiO_2_ nanorobots was further confirmed by their UV–visible absorbance spectra (Figure 2G). Colloidal Au NPs with particle sizes less than 5 nm exhibited a characteristic surface plasmon resonance (SPR) peak at 514 nm as reported, which remained unchanged after the assembly of the Au-SiO_2_ nanorobots.^25^ X-ray photoelectron spectroscopy (XPS) analysis further indicates the presence of Au NPs in their metallic state attached to silicon oxide (Figure S1). A negligible amount of carbon from adventitious carbon contamination can be observed. By adjusting the mixing ratio of Au NPs to NH_2_–SiO_2_ NPs, we successfully assembled Au-SiO_2_ nanorobots with heterogeneous Au NPs anchored onto the SiO_2_ NPs.

### Generation of Steady-State H_2_O_2_ from Glucose Catalyzed by Au-SiO_2_ Nanorobots

Both theoretical and experimental evidence indicate that Au NPs catalyze the oxidation of glucose via a two-electron transfer pathway, producing H_2_O_2_.^23,27^ However, similar to other noble metal nanoparticles such as platinum and silver, Au NPs have been reported to catalyze the decomposition of H_2_O_2_ into water and oxygen.^22^ This cascading reaction (eqs 1 and 2) where a single catalyst controls both the production and decomposition of a molecule, leads to interesting chemical dynamics (Figure 3A).

1 $$\mathrm{Glucose}+O_{2}\to \mathrm{Glucono}‐\delta ‐\mathrm{lactone}+H_{2}O_{2}$$

2 $$2H_{2}O_{2}\to 2H_{2}O+O_{2}$$

**Figure 3:**
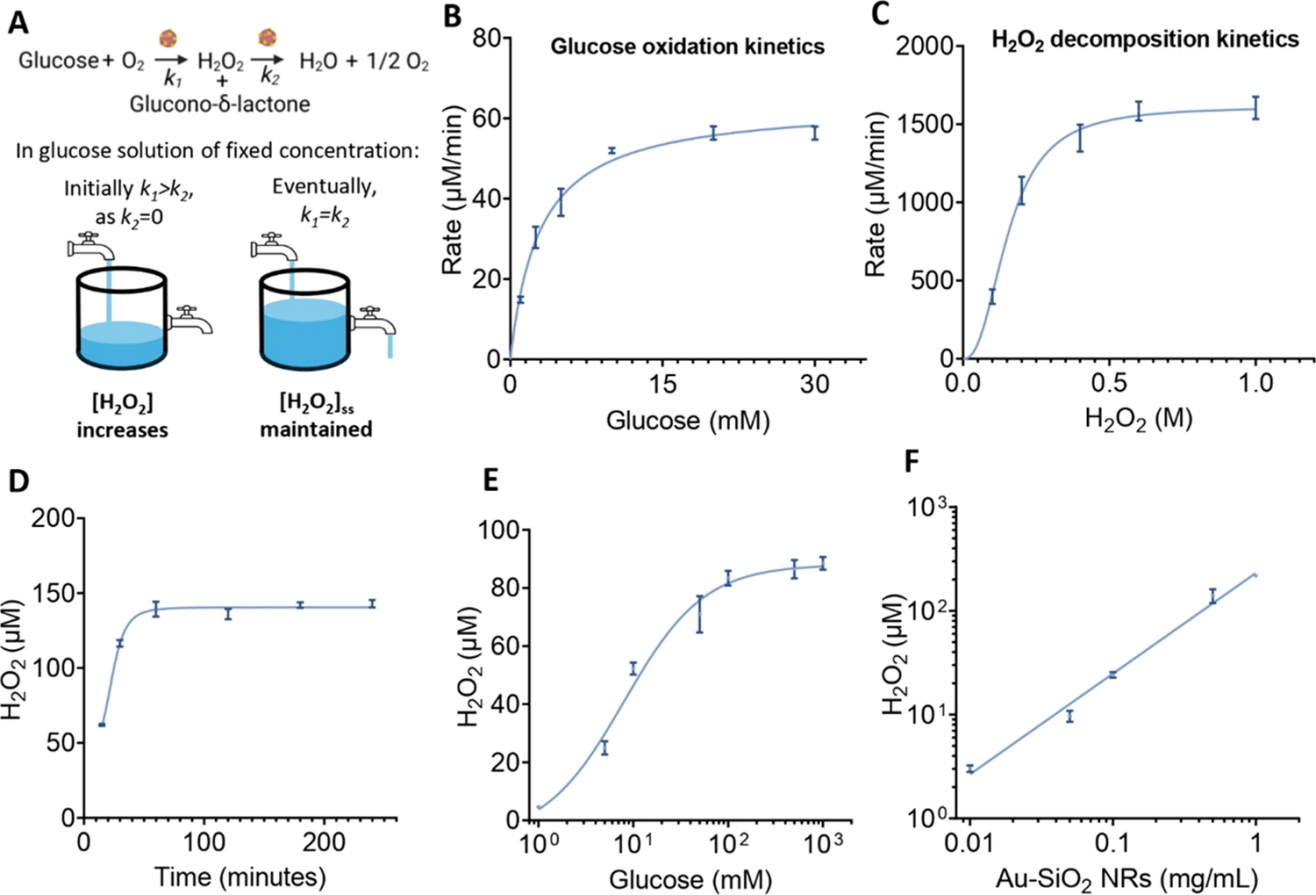
The mechanism of steady-state H_2_O_2_ generation catalyzed by Au-SiO_2_ nanorobots. (A) Schematic representation showing the cascade reaction occurring on the surface of Au-SiO_2_ nanorobots. The mechanism of the steady-state generation is demonstrated schematically and detailed in the text. (B) Michaelis–Menten kinetics for glucose oxidation catalyzed by 0.1 mg/mL Au-SiO_2_ nanorobots. (C) Sigmoidal kinetics for H_2_O_2_ decomposition catalyzed by 0.1 mg/mL Au-SiO_2_ nanorobots. (D) The steady-state concentration of H_2_O_2_ catalyzed by 0.5 mg/mL Au-SiO_2_ nanorobots in 5 mM glucose solution over 4 h. (E) Changes in the steady-state H_2_O_2_ concentration with varying concentrations of glucose with 0.1 mg/mL Au-SiO_2_ nanorobots after 1 h. (F) Changes in the steady-state H_2_O_2_ concentration with varying concentrations of Au-SiO_2_ nanorobots in a 5 mM glucose solution after 1 h. (B–F) Error bars represent standard deviation.

Characterization of this dynamic process was explored by separate saturation kinetics studies for both glucose oxidation and H_2_O_2_ decomposition by Au-SiO_2_ nanorobots. For this, the rate of substrate consumption was measured as a function of the substrate concentration (Figure 3B and C). Both glucose oxidation and H_2_O_2_ decomposition catalyzed by Au-SiO_2_ nanorobots were observed to follow enzyme-like kinetics. While glucose oxidation followed the typical hyperbolic Michaelis–Menten kinetics, the peroxide decomposition kinetics showed a sigmoidal response, typically expressed by allosteric enzymes and certain nanozymes.^28^ Critically, however, the Michaelis–Menten constant (*K*_m_) and its analog for allosteric kinetics (*K*_0.5_), for the two reactions were different by 2 orders of magnitude. For the glucose oxidation reaction, a *K*_m‑glucose_ of 2.9 mM was recorded while for H_2_O_2_ decomposition *K*_0.5–H_2_O_2__ was observed to be significantly higher at 155.1 mM.

In the presence of glucose, the reaction cascade catalyzed by Au-SiO_2_ nanorobots also leads to interesting dynamics regarding the steady-state concentration of H_2_O_2_ measured in the bulk solution. In 5 mM solutions of glucose with 0.5 mg/mL Au-SiO_2_ nanorobots, a stabilized H_2_O_2_ concentration was reached within 1 h of reaction time (Figure 3D). Moreover, this steady-state concentration of H_2_O_2_ can be influenced in two ways. First, by changing glucose concentration (Figure 3E), we observe an increase in the steady-state concentration of H_2_O_2_. However, as can be expected from the low value of *K*_m‑glucose_, Au-SiO_2_ nanorobots show substrate saturation at low glucose concentrations in the mM range and as a result, any subsequent increase in substrate concentration had little effect on the rate of H_2_O_2_ generation and the final steady-state H_2_O_2_ concentration.

Second, the steady-state concentration can also be influenced by changing concentration of Au-SiO_2_ nanorobots. Interestingly, at fixed glucose concentrations, we observe a linear relationship between the concentration of Au-SiO_2_ nanorobots and the steady-state H_2_O_2_ concentration (Figure 3F). This behavior is attributed to the difference in saturation kinetics of the two reactions. The steady-state concentration of H_2_O_2_ results from the balance between its production and decomposition, which can be mathematically approximated. Assuming a constant supply of glucose and oxygen, H_2_O_2_ production follows pseudozero-order kinetics, represented by rate constant *k*_1_. Meanwhile, its decomposition follows first-order kinetics, represented by rate constant *k*_2_, because the rate of H_2_O_2_ breakdown depends directly on its concentration (Figure 3A). Thus, the overall change in H_2_O_2_ concentration over time can be expressed as

3 $$d[H_{2}O_{2}]/\mathrm{dt}=k_{1}-k_{2}\left[H_{2}O_{2}\right]$$

At a steady-state, where production and decomposition are balanced, *d*[H_2_O_2_]/*dt* = 0, the concentration of H_2_O_2_ stabilizes to [H_2_O_2_] = *k*_1_/*k*_2_.^17,19^

Intuitively, given that both reactions are catalyzed by the same catalyst, an increase in the concentration of the catalyst is expected to increase both reaction rates by an equivalent factor and therefore not influence the steady-state concentration. However, there is an observed linear relationship between the catalyst concentration and the final produced steady-state H_2_O_2_ concentration. This observation is expected if one of the reactions occurs at or near saturation of the catalyst. In this case, when the nanorobots are exposed to a glucose concentration of 5 mM, analogous to the approximate conditions *in vivo*, the glucose oxidation reaction that produces H_2_O_2_ occurs above the *K*_m‑glucose_ of the catalyst, therefore the rate of the reaction is limited by the concentration of nanorobots. On the other hand, the H_2_O_2_ decomposition reaction occurs far from saturation and the rate of this reaction is limited by the concentration of substrate. Therefore, in this system, increasing the amount of Au-SiO_2_ nanorobots significantly influences the rate of H_2_O_2_ production (*k*_1_) while the rate of H_2_O_2_ decomposition (*k*_2_) lags behind. Consequently, the steady-state concentration of H_2_O_2_ can be controlled directly by controlling the concentration of Au-SiO_2_ nanorobots.

### Glucose-Fueled Enhanced Diffusion of Au-SiO_2_ Nanorobots

Historically, many catalytic micro/nanorobots used fuels such as H_2_O_2_ or hydrazine, which provide effective propulsion but raise significant concerns about toxicity, especially in biological applications.^29,30^ Toxic byproducts from these fuels limit the practical application of such systems within living organisms, where compatibility with cellular environments is essential. This challenge has spurred the exploration of alternative, biocompatible fuels, such as glucose, which is abundant in biological environments and presents a safer, nontoxic option.^16^

Recent advances have shown promise in using glucose oxidation and a cascading combination of glucose oxidation and H_2_O_2_ decomposition to power nanorobots, leveraging enzyme-catalyzed reactions as in the case of Au-SiO_2_ nanorobots to drive movement.^15,16,31–37^ To study the motion behaviors of Au-SiO_2_ nanorobots, we measured their mean squared displacement (MSD) by nanoparticle tracking analysis (NTA) using varying concentrations of glucose as fuel. NTA based on dark-field laser scattering, offers a reliable method for particle tracking under 100 nm. Compared to ensemble techniques like dynamic light scattering, which can be affected by aggregation, or fluorescence-based methods such as fluorescence correlation spectroscopy, which are prone to photobleaching and blinking artifacts, single particle tracking via dark-field laser scattering offers significantly improved reliability and resolution for detecting subtle diffusion changes.^38^ In addition, all data sets were corrected for external drift and mean squared displacement analysis was performed on over 250 drift-corrected trajectories per condition, well above the minimum required to detect small changes in diffusion.^39,40^

The MSD of tested nanorobots increased from 21.19 ± 0.23 μm^2^/s in water to 29.29 ± 0.17 μm^2^/s in a 55.5 mM glucose solution representing around 38% diffusion enhancement (Figure 4B and C). However, whether this motion is a result of glucose oxidation or rather a result of the cascade reaction where ultimately H_2_O_2_ decomposition leads to motion remains unclear. To investigate this, we first examine if Au-SiO_2_ nanorobots show enhanced diffusion with H_2_O_2_ as the sole reactant. We observe similar enhancements in the diffusion coefficient of Au-SiO_2_ nanorobots where the MSD reached 32.80 ± 0.38 μm^2^/s in a 1 M H_2_O_2_ solution, representing a 55% diffusion enhancement (Figure 4E and F).

**Figure 4:**
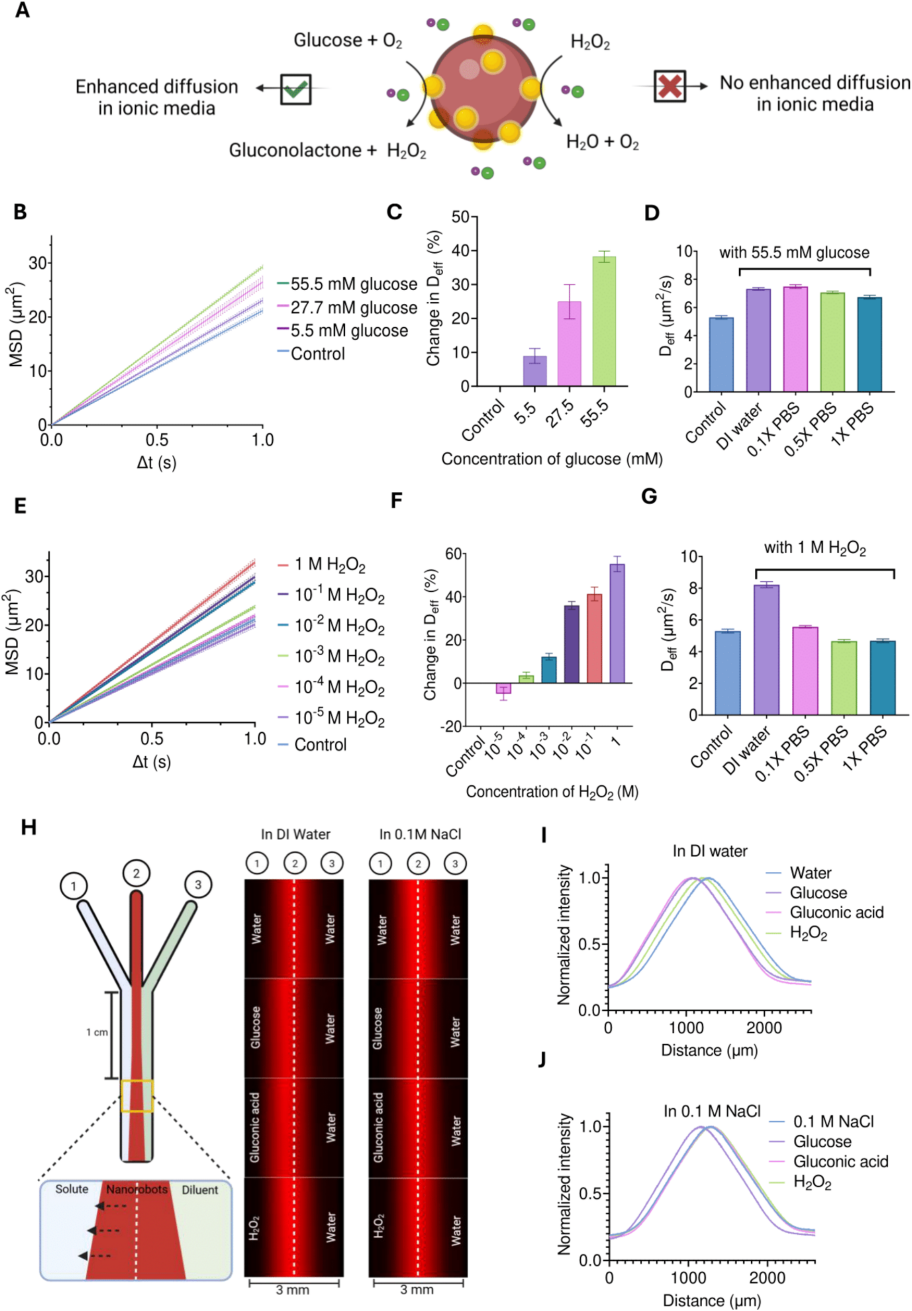
Investigation of the propulsion mechanism of glucose-powered Au-SiO_2_ nanorobots. (A) Schematic representation showing the difference in response to ionic media when using either glucose or H_2_O_2_ as a fuel. (B) The motion of Au-SiO_2_ nanorobots with varying concentrations of glucose with the linear fit of mean MSD curves. (C) Percentage change in diffusion coefficient *D*_eff_ based on glucose concentration variation. (D) Influence of ionic strength (by different concentrations of PBS solution) on the measured diffusion coefficient when using glucose as a fuel. (E) The motion of Au-SiO_2_ nanorobots with varying concentrations of H_2_O_2_ with the linear fit of mean MSD curves. (F) Percentage change in diffusion coefficient *D*_eff_ based on H_2_O_2_ concentration variation. (G) Influence of ionic strength (by different concentrations of PBS solution) on the measured diffusion coefficient when using H_2_O_2_ as a fuel. (B–G) *D*_eff_ calculated from the mean MSD fits obtained by fitting particle tracks (*N* > 250) during the first second, error bars represent 95% confidence intervals. (H) Schematic demonstrating gradient generation in a 3-inlet microfluidic channel and micrographs showing the diffusiophoretic shift. (I) Change in normalized fluorescence intensity profile with varying solute gradient in deionized water. (J) Change in normalized fluorescence intensity profile with varying solute gradient in 0.1 M NaCl solution. Schematics were created with BioRender.com.

Although at first glance, the enhanced diffusion of nanorobots fueled by glucose might seem to be caused by H_2_O_2_ produced during glucose oxidation, further investigations reveal that it is not the case. First, at low concentrations of H_2_O_2_-comparable to the steady-state levels produced by millimolar concentrations of glucose-the diffusion enhancement was negligible (Figure 4F). At an H_2_O_2_ concentration of 100 μM, the diffusion coefficient only increased by 3.6%. With the increase in H_2_O_2_ concentration, the increase of diffusion coefficient increased more profoundly. However, we cannot fully rule out this possibility, since H_2_O_2_ is produced and consumed in the vicinity of Au nanoparticles, and as a result, the local fuel concentration near the particles would be higher than the steady-state concentration measured in the bulk, leading to a higher catalytic rate comparable to that observed with higher bulk concentrations of H_2_O_2_.

Second, we examined the motion behavior of these nanorobots with glucose and H_2_O_2_ in high ionic strength media. Motion in ionic media is commonly used as a test to identify the operating mechanism of micro/nanorobots (Figure 4A). Among the common propulsion mechanisms such as bubble propulsion, neutral self-diffusiophoresis, ionic self-diffusiophoresis, and self-electrophoresis, the latter two are sensitive to the presence of ionic media and result in significant reduction of speed. Both mechanisms of ionic self-diffusiophoresis and self-electrophoresis ultimately rely on the formation of dipoles and electric fields, the presence of charged ions in the media neutralizes the charges, dampening the effect of the field and consequently reducing the phoretic effect.^41,42^ When comparing our nanorobots’ behavior in an ionic media, we observe that glucose-driven diffusion was negligibly affected by the presence of phosphate-buffered saline (PBS), whereas H_2_O_2_-driven motion was significantly suppressed (Figure 4D and G). This observation suggests two inferences: (1) the enhanced diffusion of Au-SiO_2_ nanorobots in glucose is not a result of the cascade reaction ending in H_2_O_2_ decomposition but rather due to the oxidation of glucose; and (2) different mechanisms underlie the motion driven by glucose versus H_2_O_2_, with the latter likely involving electrophoresis or ionic solute-driven diffusiophoresis.

For motion powered by glucose oxidation, the enhanced diffusion of smaller (<100 nm) nanorobots is well-documented, yet larger particles have yielded mixed results.^15,36,43,44^ Examining previous reports on glucose oxidation-driven motion in nanorobots, we note several reports of enhanced diffusion in ionic media such as PBS or cell culture media leading to inferences of neutral solute-driven self-diffusiophoresis.^15,36,37^ As the name suggests, this is a form of self-diffusiophoresis generated due to a concentration gradient of neutral molecules. Although well-established theoretically,^45,46^ its experimental demonstration in propelling colloids, to date, lacks definitive proof.^44^ This is because neutral diffusiophoresis is generated due to weak interactions between molecules and the surface of the particles such as van der Waals effect or steric exclusion effects. Simply, if the surface of the particle and the molecule in gradient experience mutually attractive or repulsive interactions, a pressure gradient forms to impose a net propulsive force on the particle.^42^ These weak effects are challenging to quantify experimentally and prone to disturbance. As a result, definitive proof of neutral self-diffusiophoretic colloids remains elusive.^47^ Additionally, the propulsive effect generated by neutral solutes dwarfs in comparison to that generated by electrostatic and ionic effects.^48^ In such a perspective, the observed small diffusion enhancement of 38% would appear to be consistent with the weak phoretic force that can be potentially produced through nonelectrolytic interactions.

To investigate which of the involved solutes can induce significant diffusiophoretic forces, we performed a series of experiments where nanorobots were exposed to externally generated concentration gradients of glucose, gluconic acid, and hydrogen peroxide. While other short-lived molecules such as gluconolactone or transient intermediates of the catalytic reaction may also contribute to self-diffusiophoresis, establishing a stable gradient of these species is experimentally challenging.

Concentration gradients were generated using a microfluidic device with three inlet channels: the solute of interest was introduced through the left channel, rhodamine-labeled nanorobots through the central channel, and water through the right channel (Figure 4H). Such microfluidic systems have been widely employed to study diffusiophoresis,^49,50^ and more recently, chemotaxis in micro- and nanorobots.^51,52^

The microfluidic setup ensures laminar flow, allowing the three streams to flow parallelly without convective mixing. However, diffusion at the interfaces between streams leads to the formation of solute gradients across the channels. The steepness of these gradients naturally decreases with increasing distance from the point of entry.^52^ Here, diffusiophoresis can be observed in the shift of the fluorescence intensity either toward the solute or away from it.

All experiments were conducted using low solute concentrations (10 mM) to minimize potential artifacts arising from convective flows.^53^ Diffusiophoretic responses were quantified by tracking the lateral shift in fluorescence intensity of the nanorobots across the channel width. As shown in Figure 4I, the largest shift was observed in response to gluconic acid (276.4 μm), followed by glucose (214.4 μm), and hydrogen peroxide (91.2 μm). In all cases, nanorobots migrated toward the analyte. This was most expected with gluconic acid since the response is likely dominated by electrolyte diffusiophoresis, as the solute was introduced in the form of sodium gluconate. Due to the substantial difference in diffusion coefficients between the larger gluconate anion and significantly smaller sodium cation, this system can generate strong ionic gradients, which can drive electrophoretic motion. As the nanorobots exhibit positive zeta potential, and as the cation (Na) diffuses faster than the anion (gluconate) the force pushes the particles up the gradient.^54^ However, for neutral solutes like glucose and H_2_O_2_, the direction of diffusiophoretic motion is difficult to predict a priori, as it depends on specific interactions between the solute and the particle surface. In this case, given the shift toward the solute in both cases, we expect a primarily attractive interaction between the particle and the solute, with a stronger interaction with glucose as compared to H_2_O_2_.

To isolate the neutral solute component of diffusiophoresis, we conducted a second set of experiments in which all solutions, including the solute, nanorobot suspension, and the right inlet stream, were prepared in 0.1 M NaCl. This ensured that no electrolyte gradients were present across the channel, effectively suppressing any contributions from ionic diffusiophoresis. Under these conditions, only glucose induced a significant lateral shift in fluorescence (142.4 μm), while gluconic acid (−17.6 μm) and hydrogen peroxide (−24.0 μm) showed minimal deviations relative to the control (Figure 4J). These results strongly suggest that nanorobot diffusiophoresis is predominantly driven by glucose gradients.

Furthermore, we explore the mechanism underlying the observed enhanced diffusion when H_2_O_2_ is used as a fuel. For nanorobots powered by H_2_O_2_, there exists a rich diversity of mechanisms with robust experimental demonstrations of self-electrophoresis and bubble propulsion.^55,56^ In addition, based on observations of catalase-powered motors where no bubbles are observed, there have also been suggestions of self-diffusiophoresis.^14,57,58^

Although we observe bubble formation at high H_2_O_2_ concentrations (1 M) for high densities of nanorobots (1 mg/mL) likely due to the rapid production of oxygen gas that exceeds the dissolved oxygen saturation point in water (Figure S2), we rule out the possibility of bubble propulsion for two reasons. First, we observe no bubble formation at low particle densities with either high concentrations of H_2_O_2_ or glucose (SI Video 1). Second, the H_2_O_2_-powered motion being sensitive to the ionic strength of media is not consistent with the mechanism of bubble propulsion which is insensitive to ionic strength.^45^

Instead, we explore the possibility of H_2_O_2_-powered Au-SiO_2_ nanorobots driven by self-electrophoresis. Self-electrophoresis requires the separation of anodic and cathodic regions on the surface of the colloid.^42^ In early designs of nanorobots, this was achieved by constructing conductive nanorobots with two different metals such as gold and platinum.^29^ However, self-electrophoretic motion was also later demonstrated for platinum (Pt)-insulator motors such as Pt-SiO_2_ and Pt-polystyrene motors, which were constructed by sputter-coating Pt onto monodisperse insulating colloids.^59^ The separation of anodic and cathodic regions is understood to develop due to a difference in reactivity across the Pt cap, this difference in reactivity consequently was attributed to be a result of the difference in thickness of Pt at the pole as compared to the edge of the catalytic cap.^55^ In the case of Au-SiO_2_ nanorobots, although no such thickness differential exists, one can imagine a variance in reactivity on the surface of a gold nanoparticle, where the part facing the silica particle has a lower reaction rate due to steric exclusion of the substrate. Whether such a difference over a sub-10 nm particle could lead to a separation of anodic and cathodic halves, however, remains an open question.

### Glucose-Powered Au-SiO_2_ Nanorobots Communicate with Cells to Induce Migration

Migration of cells is a process essential for embryonal development, proper immune response, and tissue regeneration. Despite the diversity of migratory behaviors in cell biology, studies on cell migration have revealed several common features. Migration begins with a chemical signal that triggers polarization and protrusion of a cell in the direction of movement. The direction of movement is ultimately controlled with chemical gradients of attractive or repulsive molecules. These molecules are detected by membrane proteins, the activation of which leads to a cascading process that determines the migration direction.^60,61^

Conventional methods for inducing cell migration and proliferation typically rely on the external administration of drugs or specific biochemicals, such as growth factors or chemokines. These approaches often suffer from limited spatial control, show transient activity, and affect potential off-target sites simply due to diffusion.^19,62^ H_2_O_2_ as discussed is one such molecule capable of inducing cell migration. Although migratory/proliferative effects can be induced by periodic doses of H_2_O_2_, its concentration in the system fluctuates significantly due to continuous decomposition by cellular catalase.^19,20,63^ For instance, as reported by Wagner *et al*., in human histiocytic lymphoma U937 cells exposed to 20 μM H_2_O_2_, the rate constant for H_2_O_2_ removal has been reported as approximately 10.4 × 10^–12^ s^–1^ cell^–1^ L.^64^ This rapid degradation complicates the sustained delivery of H_2_O_2_ using traditional methods.

The Au-SiO_2_ nanorobots designed in this study allow for the possibility of generating a steady-state H_2_O_2_ concentration directly *in situ*. We further studied the influence of the generated steady-state H_2_O_2_ as a biochemical signaling molecule for inducing cell migration.

The cell migration assay, or gap closure assay, a long-established standard method, is applied to investigate the influence of nanorobot treatment on collective cell migration (Figure 5A).^65^ To investigate the influence of Au-SiO_2_ nanorobots-cell communication inducing cell migration, we established a migration assay with a starting gap of 500 μm. For this study, we employed HT1080 human fibrosarcoma cells in minimum essential media with 1 g/L glucose which allows the half-reaction of glucose oxidation to take place near the *K*_m‑glucose_ of the nanorobots. Fibrosarcoma cells with their similarity to fibroblasts, are far more sensitive to H_2_O_2_ compared to keratinocytes, where previous works show signs of cytotoxicity at H_2_O_2_ concentrations as low as 10 μM.^66^ To study the effect of steady-state H_2_O_2_ concentration, we chose Au-SiO_2_ nanorobots concentrations at two dosages, a higher dose of 25 μg/mL which produces approximately 10 μM H_2_O_2_ as tested previously, and a dosage 4 times lower at 6.25 μg/mL. We note, however, that the steady-state concentration of H_2_O_2_ is lower in cell culture media when it contains cells as the intracellular catalase contributes to the rate of the H_2_O_2_ decomposition reaction (Figure S3). We first performed an MTS assay to assess the influence of the nanorobots on the metabolic activity of cells. The results showed no significant decrease in the metabolic activity of cells at 6.25 μg/mL but a 21.9% decrease at 25 μg/mL (Figure S4).

**Figure 5:**
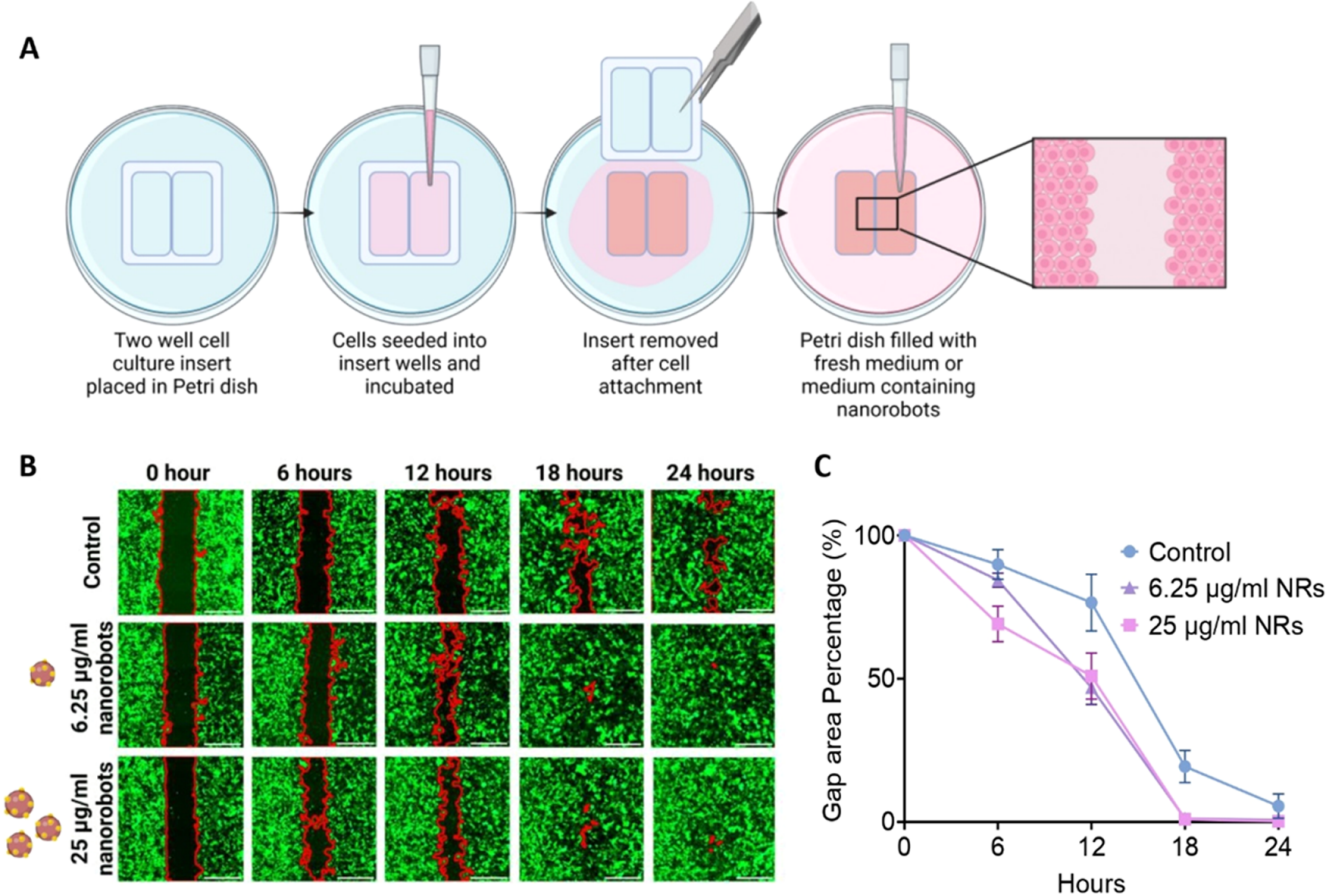
The communication of Au-SiO_2_ nanorobots with cells inducing cell migration. (A) Schematic representation of the experimental method to study the influence of the nanorobots on cell migration. (B) Confocal micrographs showing the gap closure over 24 h for nontreated cells (control) as well as nanorobot-treated cells. The scale bar is 500 μm. The red contour highlights the area of the gap, while the green color shows eGFP-expressing HT1080 cells. (C) Plotted relative gap area percentage as a function of time for control and two dosages of nanorobot-treated cells, error bars represent standard deviation of replicates. Schematic was created with BioRender.com.

The migration assay was performed over 24 h and a significant difference was observed in the Au-SiO_2_ nanorobot-treated cells when compared to untreated cells. After 12 h of incubation, the mean gap area percentage for untreated cells was observed to be 76.6 ± 9.8% while for the low and high-dose Au-SiO_2_ nanorobot treatments, the values were 46.9 ± 5.9% and 50.9 ± 8.0%, respectively (Figure 5B and C). The trend continued after 24 h, where a small gap of 5.5 ± 4.2% existed in the untreated cells while the gaps were completely closed in both nanorobot-treated samples. Although a significant difference in gap-closing speed can be observed between the treated and untreated samples, there is little difference based on the dosage of Au-SiO_2_ nanorobots after 24 h. One marked difference due to the dosage effect of Au-SiO_2_ nanorobots, however, lies at the 6-h treatment time point. The remaining gap at 6 h with the high dosage of Au-SiO_2_ nanorobots is significantly lower at 69.1 ± 6.2%, as compared to 84.3 ± 2.4% for the low dose, and this trend reversed after 12 h as noted above.

The gap-closing rate is influenced by two factors, primarily the cell migration and, secondarily, the cell proliferation rate. If certain treatment affects the rate of cell proliferation, one might expect minimal differences in the rate of gap closure during time frames shorter than the cell doubling time. In such cases, significant differences would only become apparent in longer-term experiments.^67^ Thus, at this point, it is difficult to differentiate the mechanism of cell-nanorobot communication, where such observation is due to nanorobots inducing cell migration, altering cell proliferation time, or both.

### Differentiation between Cell Migration and Cell Proliferation by Quantitative Phase Imaging

To distinguish between nanorobot-induced cell proliferation and cell migration, we conducted a series of experiments using a cell “random walk” assay where we directly observed the growth of a population of cells monitoring their motion and proliferation rates (Figure 6A).^68^ For this, we employed holographic incoherent-light-source-quantitative phase imaging (Hi-QPI) technique which allows direct observation of unstained living cells as well as quantitative dry mass measurements of cells to monitor cell growth (SI Video 2).^69^

**Figure 6:**
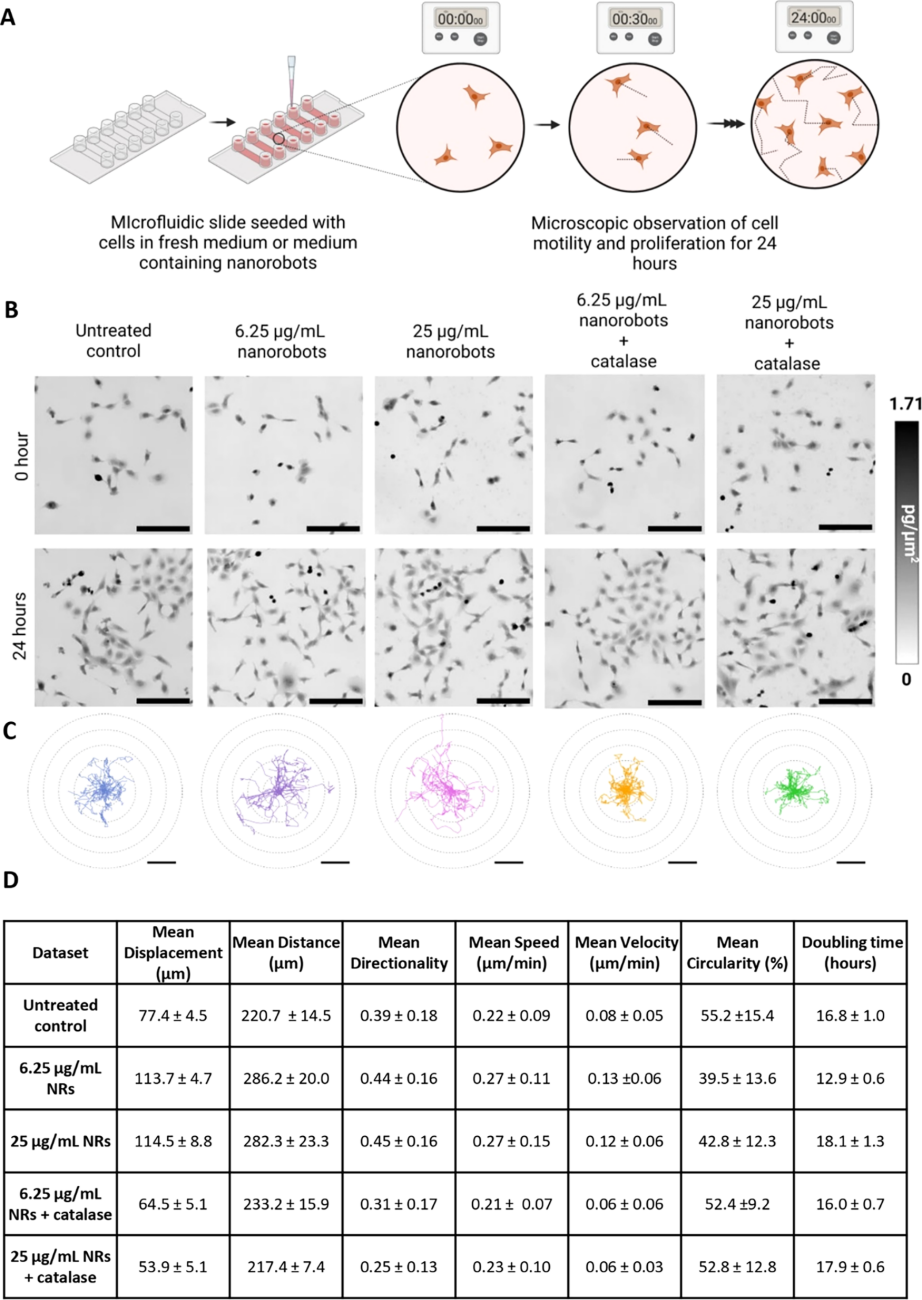
Differentiation between cell migration and cell proliferation using quantitative phase imaging. (A) Schematic illustration showing the experimental workflow of the “random walk” assay. (B) Compensated phase micrographs of the initial and final distribution of cells (scale bar is 200 μm) (C) Motility tracks of cells with and without nanorobot treatment plotted from a common origin. (Scale bar is 100 μm) (*N* = 30). (D) Table of motility parameters for all studied populations, error values show standard error of mean for mean displacement and mean distance, 95% confidence interval for doubling time and standard deviation for all other parameters. Schematic was created with BioRender.com.

In addition, to confirm that the influence of the nanorobots is through the steady-state H_2_O_2_ produced, we perform random walk assays with the inclusion of enzyme catalase combined with the nanorobot treatment. By cotreating cells with catalase and nanorobots, we aimed to selectively eliminate the H_2_O_2_ signal and assess whether the observed cellular responses were indeed H_2_O_2_-dependent.

Time-lapse imaging was employed to observe the effects of two concentrations of Au-SiO_2_ nanorobots, with and without enzyme catalase, on sparsely seeded HT1080 cells at 30 min intervals over 24 h (Figure 6B). While no significant change in the dry mass doubling time was observed, marked differences in cell motility were evident between the untreated and catalase-treated cells compared to the nanorobot-treated groups. Untreated and catalase-treated cells remained within 100 μm of their original position, whereas low-dose nanorobot-treated cells migrated over 150 μm, and those treated with the high-dose nanorobots traveled over 200 μm (Figure 6C).

In addition, we observed consistent trends indicative of heightened mobility in nanorobot-treated cells across all motility parameters (Figure 6D). Treatment with Au-SiO_2_ nanorobots led to a ∼1.2 to 1.5-fold increase in both average displacement and total distance traveled, along with higher directionality and a noticeable rise in both speed and velocity. These enhancements were observed at both tested nanorobot dosages. This increase in motility was also accompanied by noticeable changes in cell morphology (Figure S6). Specifically, nanorobot-treated cells exhibited a decrease in mean circularity, adopting more elongated and polarized shapes, consistent with a migratory phenotype.^70^

In contrast, the addition of catalase markedly suppressed cell motility across all parameters, supporting the role of nanorobot-generated H_2_O_2_ in promoting directional cell migration.

A significant difference was also observed in cell proliferation between the low- and high-dosage nanorobot treatments. The mean doubling time of cells decreased from 16.8 h in the untreated group to 12.9 h in the low-dose group (77% of the control) but increased to 18.1 h in the high-dose group (108% of the control). Notably, the addition of catalase to the low-dose treatment restored the cell doubling time to 16 h, similar to the untreated control, while catalase had little effect on the high-dose treatment. This residual effect in the high-dose group might stem from mechanisms independent of H_2_O_2_, potentially due to nanorobot-induced metabolic competition (e.g., for glucose) or other subtle interactions of the catalytically active gold nanoparticles with the cellular environment.

We infer that Au-SiO_2_ nanorobots, through steady-state generation of H_2_O_2_, influence not only cell migration but also cell proliferation. At lower doses, nanorobots enhance both migration and proliferation, whereas at higher doses, the proliferative effect is diminished. The Au-SiO_2_ nanorobots described in this study function as autonomous nanoreactors that continuously catalyze the conversion of glucose into H_2_O_2_ *in situ*. This results in the generation of a steady-state biochemical signal that is physiologically relevant and sustained, mimicking the natural steady-state already existing in cells. The localized and self-regulated generation of H_2_O_2_ allows for precise modulation of the cellular microenvironment without the need for external stimuli or complex delivery systems.

The role of H_2_O_2_ and ROS molecules as a migration-inducing molecule has been well documented across cell types.^71^ Initial studies suggest that ROS could promote cell migration by showing that ROS-degrading enzymes reduced the migration of endothelial, neutrophil, fibroblast, and muscle cells toward growth factors and chemoattractants.^72–75^ Hydrogen peroxide has been found to enhance cell migration, as its breakdown hindered chemoattractant-induced migration.^75^ Further research with keratinocytes and keratinocyte-fibroblast coculture models also shows that the addition of external bolus doses of 500 μM H_2_O_2_ induced keratinocyte migration.^63,76^ *In vivo* experiments with a mice model showed that bolus doses of H_2_O_2_ (10 mM) enhanced angiogenesis and wound healing.^11^ Of the reactive oxygen species, hydrogen peroxide has the stability required to establish concentration gradients and exists at a significant steady-state concentration.^13,77^ This property allows H_2_O_2_ to play a key role as a wound detection system, attracting immune cells to wound sites in zebrafish, with similar effects seen in mammalian endothelial cells in culture.^78^ H_2_O_2_ is also understood to promote wound closure by enhancing not only migration but also endothelial cell proliferation.^11,63,76,79^ In this context, the dose-dependent effect we observed in this study, where low nanorobot dosage supported both migration and proliferation while a higher dosage primarily enhanced migration, is consistent with the literature on ROS sensitivity.

The role of hydrogen peroxide in promoting cell migration remains unclear, but it is understood that its effects are mediated through selective post-translational modification of proteins. One key pathway involves hydrogen peroxide acting as a redox signal, primarily by oxidizing thiol groups on cysteine residues of proteins.^80^ This oxidation can occur directly, with hydrogen peroxide reacting to form sulfenic acid at the protein thiol, which may then lead to the formation of disulfides with other protein thiols.^71^ Alternatively, hydrogen peroxide can also indirectly modify target proteins through the oxidation of redox-sensitive proteins like peroxiredoxins, which act as intermediaries in the signaling cascade. These modifications influence protein function and contribute to changes in cell migration.^71,81^

Furthermore, hydrogen peroxide may modulate redox-sensitive signaling pathways that regulate cytoskeletal dynamics, cell adhesion, and motility. Oxidative stress induced by H_2_O_2_ can also affect integrin signaling,^82^ focal adhesion turnover,^83^ and the activation of matrix metalloproteinases,^84^ all of which are crucial for cell movement. H_2_O_2_ may also impact transcription factors like NF-κB,^85^ which control the expression of genes involved in migration. However, due to the nonspecific nature of cysteine oxidation and the broad range of proteins it affects, pinpointing a specific molecular target for H_2_O_2_’s role in migration remains challenging. As a result, the full molecular mechanisms underlying its effects on cell migration continue to be an active area of research.

## Conclusions

In this study, we developed glucose-powered gold-silica (Au-SiO_2_) nanorobots that can communicate with cells to induce cell migration. We successfully synthesized Au-SiO_2_ nanorobots capable of generating a steady-state concentration of H_2_O_2_ in the presence of glucose. These Au-SiO_2_ nanorobots show enhanced diffusion in both glucose and H_2_O_2_ solutions, and experiments carried out in ionic media further reveal that the underlying mechanisms driving phoresis in the two reactions may be different with the former dependent on neutral solute interactions and the latter likely relying on charged interactions.

Importantly, the study highlights the ability of Au-SiO_2_ nanorobots to generate localized, steady-state H_2_O_2_ concentrations utilizing glucose as a fuel. This approach provides a steady source of low concentrations of hydrogen peroxide in the vicinity of cells. Our investigation into the effects of these glucose-powered Au-SiO_2_ nanorobots on cell migration and proliferation, particularly in HT1080 cells, reveals a dose-dependent response to the steady-state H_2_O_2_ levels. Low doses promoted cell motility and proliferation, while higher doses exhibited hindered proliferation while still promoting cell motility. The generated H_2_O_2_ acts as a critical signaling molecule, enabling nanorobots to communicate with cells by influencing cell behavior such as migration and proliferation.

Glucose powered nanomotors have previously utilized gold nanoparticles for glucose oxidation; however, the decomposition of the resulting hydrogen peroxide in such systems typically involved additional catalytic components such as platinum nanoparticles.^36,86,87^ In contrast, the nanorobots presented in this work employ gold nanoparticles as the sole catalytic entity capable of driving both glucose oxidation and hydrogen peroxide decomposition.

Moreover, while earlier studies aimed to eliminate H_2_O_2_ altogether to minimize cytotoxicity, our findings demonstrate that under controlled conditions, a nonzero steady-state concentration of H_2_O_2_ can be established and maintained. This persistent presence of H_2_O_2_ functions as a biochemical signal that promotes directed cell migration and proliferation, offering a mechanism for nanorobot–cell communication. Furthermore, the tunability of this steady-state concentration via nanorobot dosage introduces the potential for programmable collective behavior such as quorum sensing, logic-gated actuation, or dynamic feedback regulation.^88^ These capabilities allow for the design of responsive and autonomous nanorobotic systems for therapeutic and tissue engineering applications.

Future research could focus on validating these systems in complex tissue models. Overall, glucose-powered Au-SiO_2_ nanorobots represent a significant step toward biointegrative nanotechnology, capable of interfacing with cellular systems to influence behavior and therapeutic outcomes in a controlled manner.

## Materials and Methods

### Chemicals

Gold­(III) chloride trihydrate (HAuCl_4_·3H_2_O, 99.9%), sodium borohydride (NaBH_4_, 99%), triethoxylpropylaminosilane functionalized silica nanoparticle dispersion in water (<50 nm by DLS), d-(+)-Glucose (≥99.5%), hydrogen peroxide solution (H_2_O_2_, 30% w/w in H_2_O), rhodamine B isothiocyanate—mixed isomers, gluconic acid sodium salt (≥99%), sodium chloride (NaCl, 99.0%) and catalase from bovine liver (2000–5000 units/mg) were purchased from Sigma-Aldrich (Merck, Germany). Minimum essential medium (MEM) no glutamine, sodium pyruvate (100 mM), l-glutamine (200 mM), MEM nonessential amino acids solution (100×), fetal bovine serum (FBS), gentamicin (10 mg/mL), *N*-2-hydroxyethylpiperazine-*N*-2-ethanesulfonic acid (HEPES, 1 M) and phosphate buffered saline (10× PBS, pH 7.4) were purchased from ThermoFischer Scientific. The deionized (DI) water used in all experiments was purified in a qualified purification system.

### Synthesis of Gold Nanoparticles

Gold nanoparticles were synthesized according to a previously established method.^25^ Briefly, 1.5 mg of HAuCl_4_·3H_2_O was dissolved in 33 mL of water under constant stirring. One mL of freshly prepared NaBH_4_ solution (1.5 mg/mL) was added dropwise to the solution. The reaction mixture was stirred for 1 h at room temperature, resulting in the formation of gold nanoparticles.

### Synthesis of Au-SiO_2_ Nanorobots

For the synthesis of Au-SiO_2_ nanorobots, the particle concentrations of both Au NPs and SiO_2_ NPs were measured using dynamic light scattering instrument Zetasizer Ultra (Malvern Panalytical, U.K.). The concentrations of both solutions were adjusted to achieve a 10-fold excess of Au NPs relative to SiO_2_ NPs. The solutions were then mixed and incubated for 10 min followed by which the solution was centrifuged (Sorvall LEGEND X1, Thermo Fischer Scientific, USA) to remove the unbound gold nanoparticles. The resulting pellet was washed three times with deionized water and resuspended in 10 mL of deionized water. The suspension was sonicated for 30 min to ensure stability and uniformity, and its concentration was measured gravimetrically and adjusted to 3 mg/mL.

### Material Characterization

Morphological characterization of the Au-SiO_2_ nanorobots and their components was carried out utilizing a high-resolution scanning electron microscope Verios 460L (FEI, USA). Samples at appropriate dilutions in water were drop-casted onto holey carbon-covered copper TEM grids coated with ultrathin carbon membrane (Agar Scientific, U.K.) and imaged using a retractable STEM detector with BF/DF/HAADF segments. Hydrodynamic diameter and zeta potential measurements were performed in water using a Zetasizer Ultra instrument (Malvern Panalytical, U.K.). Visible light absorption spectra were measured using a Jasco V-750 UV–visible absorption spectrophotometer (JASCO, Japan). Surface chemical composition was analyzed with the Kratos Analytical Axis Supra XPS instrument (Kratos, Shimadzu, Japan) with a monochromatized Al Kα (1486.7 eV) excitation source. All acquired spectra were calibrated to the adventitious C 1*s* peak at 284.8 eV and fitted using KolXPD (kolibrik.net).

### Glucose Oxidation Kinetics

The kinetics of glucose oxidation catalyzed by Au-SiO_2_ nanorobots were examined by incubating Au-SiO_2_ nanorobots (0.1 mg/mL) with 1 mL of glucose solutions at various concentrations (1, 2.5, 5, 10, 20, and 30 mM) in PBS for 3, 6, and 9 min. After incubation, the suspension was centrifuged (Sorvall LEGEND X1) at 14,000 rpm for 1 min to isolate the Au-SiO_2_ nanorobots. A 100 μL aliquot of the supernatant was collected, and the glucose in the supernatant was quantified by a hexokinase assay (GAHK20, Sigma-Aldrich) by measuring absorbance at 340 nm (Jasco V-750 UV–vis spectrophotometer). Glucose consumption was calculated based on the decrease in glucose concentration, and initial reaction rates were fitted to generate a Michaelis–Menten curve following the equation:

4 $$v=\frac{V_{\max}\times \left[S\right]}{K_{m}+\left[S\right]}$$

where *v* is the reaction rate, *V*_max_ is the maximum rate, [*S*] is the substrate concentration, and *K*_m_ is the Michaelis–Menten constant.

### H_2_O_2_ Decomposition Kinetics

Similarly, H_2_O_2_ decomposition kinetics were studied by incubating Au-SiO_2_ nanorobots (0.1 mg/mL) with 1 mL of H_2_O_2_ solutions at various concentrations (0.1, 0.2, 0.4, 0.6, and 1.0 M) in PBS for 3, 6, and 9 min. After incubation, the particles were separated by centrifugation at 14,000 rpm for 1 min, and 100 μL of the supernatant was collected. The decrease in H_2_O_2_ concentration was measured using the FOX assay (MAK311, Sigma-Aldrich) by measuring absorbance at 520 nm. The reaction rates were calculated, and the plot was fitted with allosteric sigmoidal kinetic model following the equation:

5 $$v=\frac{V_{\max}\times {\left[S\right]}^{h}}{{K_{0.5}}^{h}+{\left[S\right]}^{h}}$$

where *v* is the reaction rate, *V*_max_ is the maximum rate, [*S*] is the substrate concentration, and *K*_0.5_ is the substrate concentration at half *V*_max_ and h is the Hill slope.

### Steady-State H_2_O_2_ Generation Studies

The effect of glucose concentration on steady-state H_2_O_2_ generation was assessed by incubating Au-SiO_2_ nanorobots (0.1 mg/mL) with glucose concentrations ranging from 1 to 1000 mM. H_2_O_2_ generation was measured after 1 h using the FOX assay. Additionally, the influence of nanorobots’ concentration on H_2_O_2_ production was examined by incubating nanorobots at concentrations ranging from 0.01 to 1 mg/mL. H_2_O_2_ concentrations were determined as described in the previous section.

### Motion Studies

Motion studies were conducted using a NanoSight LM14 instrument (Malvern Panalytical, U.K.) equipped with a scientific CMOS camera. The Au-SiO_2_ nanorobots were diluted to a concentration of 10^6^ particles/mL with deionized water. Glucose or H_2_O_2_ was added to final concentrations of 5.5, 27.7, and 55.5 mM for glucose, and 10 μM to 1 M for H_2_O_2_. Additional experiments were performed in 0.1×, 0.5×, and 1× PBS buffer to study the influence of ionic strength influence on the motion. Control experiments were performed in deionized water without substrates. Videos of particle motion were recorded at 25 fps for 60 s, and particle tracking was conducted using ImageJ^89^ with the TrackMate plugin.^90^ The mean squared displacement (MSD) of at least 250 tracks per concentration was analyzed in MATLAB using package MSDanalyzer^91^ for drift correction, and the MSD curve was fitted with a linear function to determine diffusion coefficients.^40^

### Microfluidic Studies

Microfluidic diffusiophoresis experiments were performed using Ibidi μ-Slide III 3in1 channel slides. Fluorescent Au-SiO_2_ nanorobots were prepared by labeling the particles with Rhodamine B isothiocyanate. Briefly, 60 mg of Au-SiO_2_ particles were incubated with 5 mg of Rhodamine B isothiocyanate in 20 mL of deionized water for 24 h. After incubation, the particles were thoroughly washed and resuspended at a concentration of 1 mg/mL either in deionized water or 0.1 M NaCl, depending on the experimental condition.

For flow control, two syringe pumps (Harvard Apparatus Pump 11 Elite) were used: a single-syringe model for introducing fluorescently labeled nanorobots and a dual-syringe model for infusing either water or solute. Both pumps were operated at a flow rate of 500 μL/h.

Fluorescence imaging was carried out using a Nikon Eclipse Ti2–U microscope equipped with a 10× objective and a Hamamatsu ORCA-Flash4.0 camera. Measurements were taken at the 1 cm position along the channel, and a 3.2 × 3.2 mm^2^ region was imaged. Within this area, a central grid measuring 5 × 10^6^ μm^2^ was analyzed to extract averaged intensity profiles. This approach was preferred to minimize the influence of noise and fluctuations caused by individual particles, which are typically more evident in standard line profiles.

### Cell Culture

Two variants of HT1080 fibrosarcoma cells were used in these experiments: unstained HT1080 cells for the “random walk” assay (obtained from CLS Cell Lines Services GmbH, Germany) and HT1080 cells permanently transfected with pcDNA3-EGFP-CAAX and p750-mRuby2-NLS plasmids to express green membrane-targeted EGFP and nuclear-localized mRuby2 for the migration assay (kind gift from Jan Brábek, Department of Cell Biology, Charles University, Prague). The cells were maintained at 37 °C in a humidified incubator with 5% CO_2_ in minimum essential medium (MEM) supplemented with nonessential amino acids (0.1 mM), 10% fetal bovine serum, sodium pyruvate (1 mM), l-glutamine (2 mM), and gentamicin (10 μg/mL).

### Cell Metabolic Activity Assay

The influence of nanorobots on the cell metabolic activity was measured by incubating 5000 cells/mL in a 96 well-plate and mixing with nanorobots at varying concentrations between 1 and 100 μg/mL for 24 h. The media containing nanorobots was replaced with fresh media containing 20 μL of CellTiter 96 AQueous One Solution (Promega) and the absorbance at 490 nm was recorded 4 h later. The metabolic activity was analyzed based on protocols of the CellTiter 96 Aqueous kit.

### Migration Assays

HT1080 cells expressing EGFP and mRuby2 were seeded at 500,000 cells/ml in 2-well Ibidi culture inserts placed in μ-Dishes (35 mm) treated with IbiTreat (Ibidi, Germany). The inserts were removed after 24 h, and fresh media containing 20 mM 4-(2-hydroxyethyl)-1-piperazineethanesulfonic acid (HEPES) (pH 7.4) and nanorobots (6.25 or 25 μg/mL) were added. Cells were imaged every 6 h for 24 h using a Zeiss LSM 980 confocal microscope (Zeiss, Germany) with a Zeiss 10× Plan-Apochromat objective. Images were acquired using an excitation wavelength of 482 nm and a detection window of 491–656 nm. Images were analyzed using ImageJ plugin wound healing size tool.^92^

### “Random Walk” Assays with Holographic Incoherent-Light-Source-Quantitative Phase Imaging (Hi-QPI)

Quantitative phase imaging (QPI) was performed using a multimodal holographic Q-PHASE microscope (Telight a.s., Brno, CZ). The unstained HT1080 cells were seeded at 60,000 cells/ml in Ibidi μ-Slide VI 0.4 chambers and incubated for 24 h. Following incubation, fresh media with 20 mM HEPES and nanorobots (6.25 or 25 μg/mL) were added. In specific experimental conditions, 100 U of catalase was also included in the media to scavenge the H_2_O_2_ generated by the nanorobots. For the duration of the time-lapse experiments, slides were maintained at 37 °C in the microscope’s environmental enclosure. Imaging was performed with a Nikon Plan 10×/0.30 objective, capturing images every 30 min over 24 h. QPI reconstruction and cell segmentation were performed using SophiQ software (Telight a.s., Brno, CZ) with a watershed algorithm. The dry mass of cells was calculated through SophiQ according to eq 6, where *m* is the dry mass density of cell (pg/μm^2^), ϕ is the phase shift (rad) detected by the microscope, λ is the wavelength of light used (μm) and α is the specific refraction increment (0.18 μm^3^/pg).^93^

6 $$m=\frac{\phi \lambda }{2\pi \alpha }$$

For motility analysis, cells were tracked using the TrackMate plugin in ImageJ, and mean speeds were calculated. The motility plots were generated in MATLAB. For proliferation measurements, cell numbers in each frame were counted and fitted with an exponential function to calculate the doubling time.

## Supplementary Material







## Notes

X-ray photoelectron spectroscopy (XPS) analysis of Au-SiO_2_ nanorobots; visual comparison of hydrogen peroxide decomposition catalyzed by Au-SiO_2_ nanorobots; concentration of hydrogen peroxide produced by 25 μg/mL Au-SiO_2_ nanorobots in cell culture media containing cells; MTS assay showing the change in metabolism of cells after treatment with different concentrations of Au-SiO_2_ nanorobots; the doubling time of cell dry mass treated with Au-SiO_2_ nanorobots at different concentrations; distribution of circularity of cells for cells treated with Au-SiO_2_ nanorobots (PDF)
Motion of nanorobots in glucose (55.5 mM) and hydrogen peroxide (1 M) (MP4)
“Random walk” assay with holographic incoherent-light-source-quantitative phase imaging (MP4)
