# Supplementary material for: Nanorobot-Cell Communication via In Situ Generation of Biochemical Signals: Toward Regenerative Therapies
Source: ACS Nano. 2025 Jun 17;19(25):22953–67. doi: 10.1021/acsnano.5c02092 (PMC12224328; doi:10.1021/acsnano.5c02092)
Supplement: Supplementary file 1 [file nn5c02092_si_001.pdf]

Supporting Information

# Nanorobot-Cell Communication via in-situ Generation of Biochemical Signals: Towards Regenerative Therapies

*Roshan Velluvakandy*[1], *Xiaohui Ju*[1], *Martin Pumera** [1,2,3]

[1] Future Energy and Innovation Laboratory, Central European Institute of Technology, Brno University of Technology, Purkyňova 123, 61200 Brno, Czech Republic

[2]Advanced Nanorobots & Multiscale Robotics Laboratory, Faculty of Electrical Engineering and Computer Science, VSB - Technical University of Ostrava, 17. listopadu 2172/15, 70800 Ostrava, Czech Republic

[3]Department of Medical Research, China Medical University Hospital, China Medical University, No. 91 Hsueh-Shih Road, 40402 Taichung, Taiwan

Email: martin.pumera@ceitec.vutbr.cz

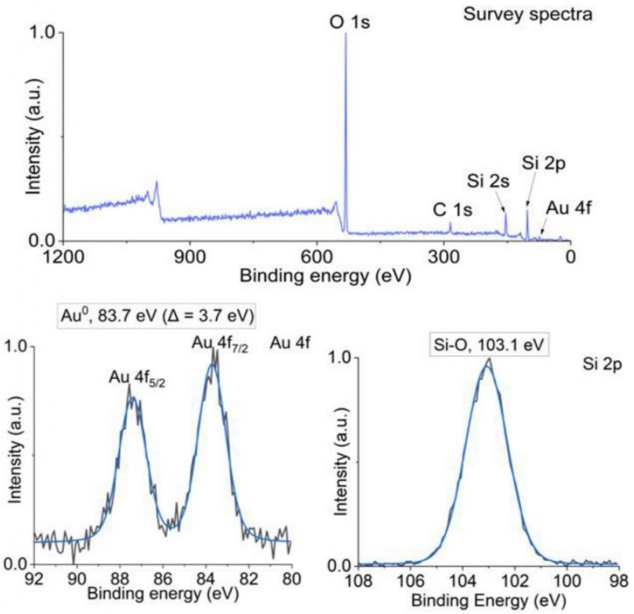


**Figure S1:** X-ray photoelectron spectroscopy (XPS) analysis of Au-$SiO_2$ nanorobots. (Top): Survey spectrum of the as-synthesized Au-$SiO_2$ nanorobots. (Bottom left): core-level spectrum of the Au 4*f* indicating the metallic state of Au NPs. (Bottom tight): core-level spectrum of the Si 2*p* indicating the presence of silicon oxide as the carrier substrate.

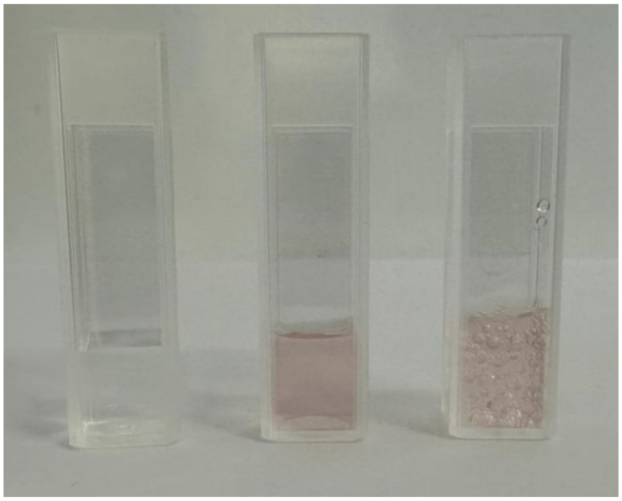

**Figure S2**: Visual comparison of hydrogen peroxide decomposition catalyzed by Au-$SiO_2$ nanorobots. (Left): Cuvette containing 1 M hydrogen peroxide solution alone, with no visible reaction. (Centre): Cuvette containing 55.5 mM glucose solution with 1 mg/ml of Au-$SiO_2$ nanorobots, showing no bubbles (Right): Cuvette containing 1 M hydrogen peroxide solution with 1 mg/ml of Au-$SiO_2$ nanorobots, showing significant bubble formation due to production of oxygen gas

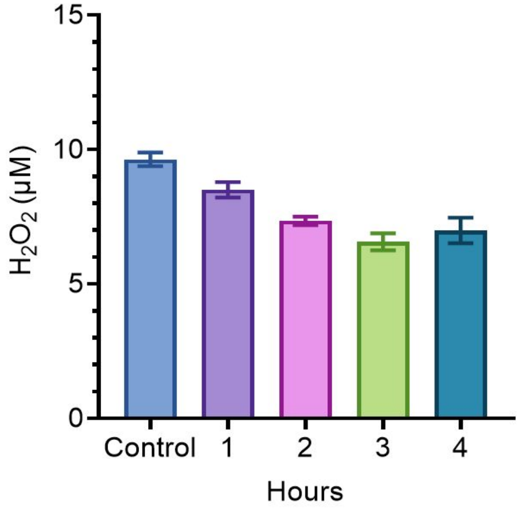


**Figure S3**: Concentration of hydrogen peroxide produced by 25 μg/ml Au-$SiO_2$ nanorobots in cell culture media containing 1,000 cells/ml. Control shows hydrogen peroxide concentration in the cell culture media without cells.

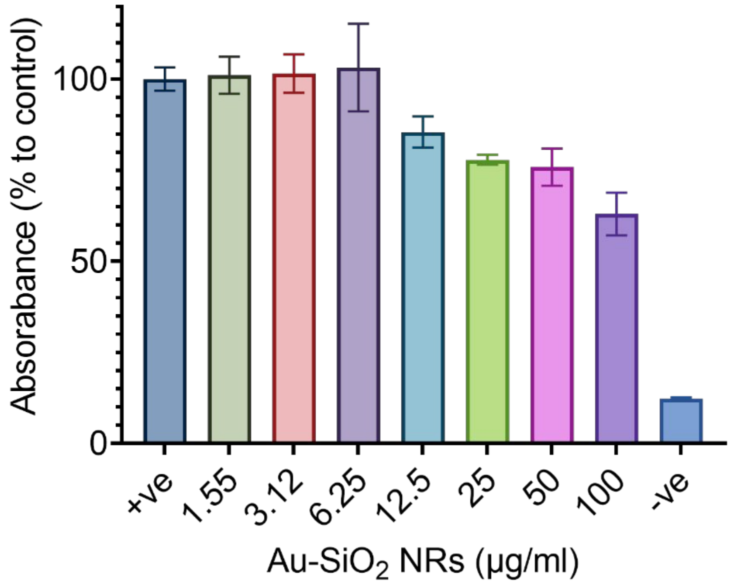


**Figure S4:** MTS assay showing the cell metabolic of cells after treatment with different concentrations of Au-$SiO_2$ nanorobots. The positive control contained cells that were not exposed to nanorobots, indicating maximum metabolic activity, while the negative control controlled no cells, indicating zero metabolic activity.

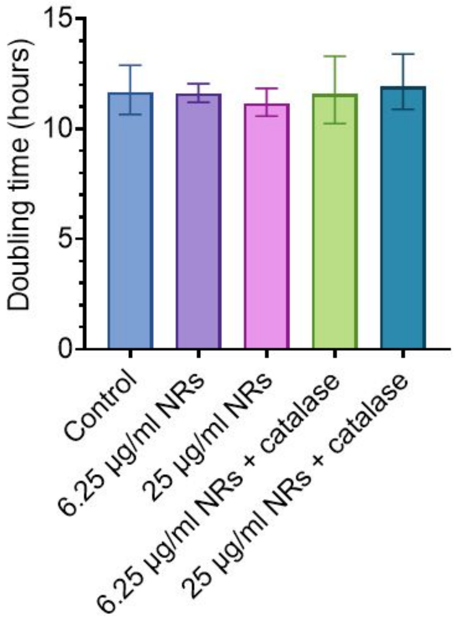


**Figure S5**: The doubling time of cell dry mass treated with Au-$SiO_2$ nanorobots at different concentrations compared to untreated cells (control) and catalase-treated cells. Dry mass was calculated using holographic incoherent-light-source-quantitative phase imaging (Hi-QPI).

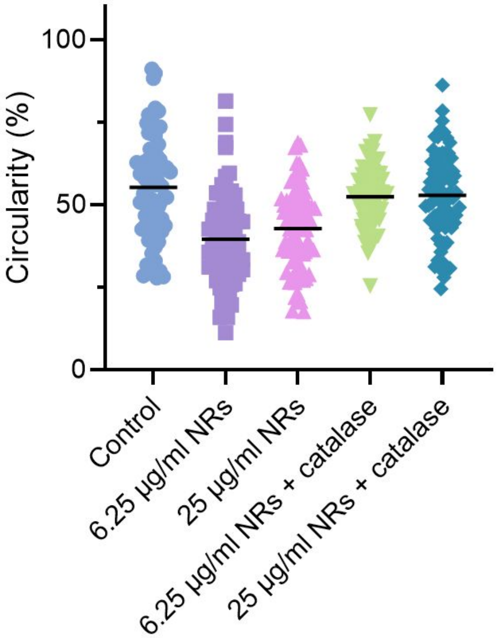


**Figure S6**: Distribution of circularity of cells for cells treated with Au-$SiO_2$ nanorobots at different concentrations compared to untreated cells (control) and catalase-treated cells.
